# Adaptation and validation of the PEDSQL™ oral health scale for toddlers in Chilean population

**DOI:** 10.1186/s12903-019-0984-1

**Published:** 2020-01-06

**Authors:** Claudia Atala-Acevedo, Carlos Zaror, Gerardo Espinoza-Espinoza, Patricia Muñoz-Millán, Sergio Muñoz, María José Martínez-Zapata, Montse Ferrer

**Affiliations:** 1 Department of Pediatric Dentistry and Orthodontics, Faculty of Dentistry, Universidad de La Frontera, Temuco, Chile; 2 Center for Research in Epidemiology, Economics and Oral Public Health (CIEESPO), Faculty of Dentistry, Universidad de La Frontera, Temuco, Chile; 3 Centro de Excelencia CIGES, Universidad de la Frontera, Temuco, Chile; 4 Department of Public Health, Faculty of Medicine, Universidad de Frontera, Temuco, Chile; 5 Iberoamerican Cochrane Centre, Biomedical Research Institute Sant Pau (IIB Sant Pau), Barcelona, Spain; 6 CIBER Epidemiología y Salud Pública (CIBERESP), Madrid, Spain; 7 Health Services Research Group, IMIM (Hospital del Mar Medical Research Institute), Barcelona, Spain

**Keywords:** Quality of life, Oral health, Child, preschool, Psychometrics

## Abstract

**Background:**

The Pediatric Quality of Life Inventory™ (PedsQL™) Oral Health Scale was developed to measure oral health-related quality of life (OHRQoL). The aim of this study was to cross-culturally adapt the parent-reported version for toddlers of PedsQL™ Oral Health Scale into Spanish and to assess the acceptability, reliability and validity of this version in Chilean preschool population.

**Methods:**

The PedsQL™ Oral Health Scale for toddlers was cross-culturally adapted for the Spanish language using the recommended standards. To assess metric properties, a cross-sectional study was carried out with 301 children aged 2 to 5 years in Carahue, Chile. Chilean versions of the PedsQL™ Oral Health Scale, PedsQL™ 4.0 Generic Core Scales, and Early Childhood Oral Health Impact Scale (ECOHIS) were completed by the children’s parents. Dental caries, malocclusion and dental trauma were examined by trained dentists. The PedsQL™ Oral Health Scale was administrated a second time 14–21 days after. The reliability of the scale was verified by analysis of internal consistency (Cronbach’s alpha) and reproducibility (Intraclass correlation coefficient – ICC). The validity of the construct was assessed by confirmatory factor analysis and known groups method. The convergent validity was assessed by calculating the Spearman’s correlation with the ECOHIS questionnaire.

**Results:**

The PedsQL™ Oral Health Scale demonstrated good reliability, with Cronbach’s alpha coefficient of 0.79 and ICC of 0.85. A moderate-to-strong correlation was found between the PedsQL™ Oral Health Scale and the ECOHIS questionnaire (− 0.64); the PedsQL™ Oral Health Scale score was lower in children with poor than those with excellent/very good oral health (median 100 vs 85, *p* < 0.001); it also was lower in children with caries than in those caries-free (median 100 vs 90, *p* < 0.001). No statistically significant differences were found among groups according to malocclusion and traumatic dental injuries.

**Conclusions:**

The PedsQL™ Oral Health Scale for toddlers in Spanish showed to be equivalent to the original version, and its psychometric properties were satisfactory for application in a Chilean pre-school population.

## Background

Oral health is an integral part of general health, and oral conditions such as dental caries, gingivitis, malocclusions or traumatic dental injuries can affect the life of an individual in areas including social, physical and emotional functioning [1–3]. For this reason, the oral health of children and adolescents may be considered particularly crucial, given that a poor state of oral health may have negative effects on their learning skills, growth, socialization and everyday activities, affecting their quality of life [4–6].

Oral Health-Related Quality of Life (OHRQoL) has been defined as “a multidimensional construct that includes a subjective evaluation of the individual’s oral health, functional well-being, emotional well-being, expectations and satisfaction with care, and sense of self” [7]. Knowledge about OHRQoL allows the perceived needs of the child and its family to be assessed [8]; it therefore helps to improve the development of oral health programs by identifying groups with higher risk, and improving access to health services [9].

In response to these potential benefits, several instruments have been developed to measure OHRQoL, determining the impact of dental diseases and treatment experiences in pre-school children. A recent systematic review identified five instruments applicable to preschoolers, the development of which was published between 2002 and 2014 [10]. Only one of them, the Pediatric Quality of Life Inventory™ (PedsQL™) Oral Health Scale, was designed to measure children’s general oral health status as a component of general health-related quality of life [11].

The PedsQL™ 4.0 questionnaire is a generic instrument for evaluating the quality of life of children and adolescents aged between 2 and 18 years, originally developed in English [12]. The version for pre-school children, which is answered by their parents and/or guardians, consists of 21 items divided into four sub-scales: Physical Functioning (8 items), Emotional Functioning (5 items), Social Functioning (5 items) and School Functioning (3 items). It was cross-culturally adapted and validated in Chile by Plaza and cols [13]., presenting adequate metric properties.

The PedsQL™ 4.0 also has specific modules for various chronic diseases and clinical situations such as asthma, arthritis, diabetes or pain, which could be used along with the PedsQL™ 4.0 Generic Core Scale. It is directed at both children suffering chronic or acute diseases and healthy ones.

One of those modules corresponds to the PedsQL™ Oral Health Scale. Few studies have assessed the psychometric properties of the PedsQL™ Oral Health Scale [14, 15], and it had not previously been cross-culturally adapted into Spanish. It is therefore of vital importance to have an OHRQoL assessment instrument linked to general health in Spanish which allows to monitor the impact of oral conditions on the quality of life of pre-school children.

The aim of this study was to cross-culturally adapt the original parent-reported version for toddlers of the PedsQL™ Oral Health Scale into Chilean Spanish and to assess the acceptability, reliability and validity of this version, which allows discriminating between groups with different oral health problems, in Chilean preschool population. Although the PedsQL™ Oral Health Scale was developed to measure OHRQoL among children aged 2 to 18 years (with parent-reported form for 2–4 years, and with parent- and self- report forms for 5–7 years, 8–12 years, and 13–18 years), we only adapted the version for toddlers because they were the priority group of Chilean oral health policies and several public health programs have been implemented in this age group.

## Materials and methods

The study was carried out in two phases. First, the version for toddlers (2–4 years old) of the PedsQL™ Oral Health Scale was translated into Spanish and adapted to the Chilean culture. In the second phase, its psychometric properties were evaluated in a Chilean pre-school sample. The study was approved by the Ethics Committee of Universidad de La Frontera (Decision 061/2015).

### Instrument

The PedsQL™ Oral Health Scale is a questionnaire containing five items with five response alternatives: 0 = never a problem, 1 = almost never a problem, 2 = sometimes a problem, 3 = often a problem, 4 = almost always a problem. The score for each item is reversed and transformed to a linear scale of 0–100 (0 = 100; 1 = 75; 2 = 50; 3 = 25; 4 = 0). The scale scores are calculated as the sum of the items divided by the number of items answered, such that a higher score indicates better OHRQoL. The version for toddlers is completed by parents or guardians [11].

### Cross-cultural adaptation

The cross-cultural adaptation of the PedsQL™ Oral Health Scale into Chilean Spanish followed international standards for the development of new linguistic versions equivalent to the original instrument [16].

The English version of the PedsQL™ Oral Health Scale was translated into Spanish independently by two professional translators whose native language was Spanish. The translators were asked to maintain the conceptual equivalence of the original version, rather than a literal translation (to obtain natural expressions which expressed the same concept), and to score the difficulty in finding the conceptual equivalence for each item from 1 (minimum difficulty) to 10 (maximum difficulty).

The two Spanish translations of the PedsQL™ Oral Health Scale were compared by a panel consisting of: two experts in OHRQoL assessment, two pediatric dentists, and the two translators. The discrepancies were discussed until a first unified version was obtained. This unified Spanish translation was reviewed by a group of parents of pre-school children (3 fathers and 4 mothers), in order to check its applicability and comprehensibility. This preliminary version was translated back into English, separately, by two native English speakers who also evaluated the difficulty in finding equivalent expressions. Finally, the equivalence between the original version and these back-translations was evaluated by the expert panel who rated the items as: A = conceptually and linguistically equivalent to the original item; B = functionally equivalent, but with grammatical differences; or C = equivalence is not obvious. Expressions evaluated with category C were reviewed by the expert panel to improve the Spanish wording to achieve equivalence. The report on equivalence between the original and back-translated versions was sent to the author of the original PedsQL™ Oral Health Scale for evaluation.

A cognitive debriefing interview was applied to 15 parents (2 fathers and 13 mothers, aged between 24 and 37 years) of children aged between 2 and 5 years, to assess the clarity and comprehensibility of the Spanish version. The parents first self-completed the whole questionnaire and afterwards, to assess what the parents had understood, they were asked open questions about their responses. A set of questions was used during the interview in order to obtain standardized information.

### Evaluation of validity and reliability

To assess the metric properties of the Chilean version of the PedsQL™ Oral Health Scale, a cross-sectional study was carried out in public preschools in the district of Carahue (Southern Chile). Eleven preschools funded by the Chilean government participated in the study, which was carried out between April and October 2016.

Pre-school children aged 2 to 5 years were included, together with their parents. The inclusion criteria were: children without any systemic diseases, long-term medication or special health needs, and that both children and parents agreed to take part in the study. Authorization was obtained through a written informed consent in the case of the parents and the verbal consent of the children. The parents were invited to a meeting in the preschool, during which the participating children had their mouths examined and parents were asked to complete three questionnaires regarding their child: the PedsQL™ 4.0 Generic Core Scale, the PedsQL™ Oral Health Scale and the Early Childhood Oral Scale (ECOHIS). The parents also completed a structured questionnaire to collect information on the child’s age, gender, socio-economic status and history of oral hygiene habits, as well as their general and dental health status. These questionnaires were sent by mail to the parents who did not attend the meeting.

Four previously trained and standardized researchers carried out the dental examinations in rooms specially equipped and adapted for the study, under artificial light, involving visual inspection of the oral cavity after prophylactic brushing with a toothbrush. The examiners were blinded to the questionnaire answers. The caries diagnosis was based on the criteria proposed by the World Health Organization (WHO) for Oral Health Surveys [17]. The types of traumatic dental injuries were classified according to Andreasen and Andreasen [18], and malocclusion was assessed by the presence or absence of at least one of the following alterations: anterior open bite, overjet> 4 mm and anterior cross-over bite [19]. The researcher standardization process consisted first of a theoretical calibration on the study protocol, diagnostic criteria, dental examination system, and filling in a clinical record. Practical calibration was then carried out on 15 children selected at random from a school located in the same district, but not included in the study. The inter-examiner agreement was high with kappa coefficients of 0.83 and 0.70 for caries and malocclusion traits. A series of 20 images was used to evaluate the reliability of traumatic dental injuries (kappa = 0.79). The global intra-examiner agreement was kappa = 0.81.

The socio-economic status of the family was estimated through the healthcare provision level determined by the state health insurance. Families were classified in the low socio-economic status if they had no resources and a taxable monthly income lower than or equal to USD 367.73.

### Statistical analysis

The sample size was estimated following the recommended standard of 2 to 20 participants per item with a minimum of 100 to 250 subjects [20, 21]. Considering this last number of participants recommended, and assuming a 20% of potential missing answers, the sample size required was of 300 children.

The descriptive analysis of the socio-demographic and clinical characteristics of the sample was carried out calculating frequencies and percentages. The feasibility and acceptability of the PedsQL™ Oral Health Scale was examined by calculating the percentage of parents who did not respond to some items. If more than 50% of the items on the scale were missing, the scale score was not calculated. When 50% or fewer items were missing, we imputed them by the mean of the completed items [11].

Distribution of the PedsQL™ Oral Health Scale scores was assessed by obtaining the range, mean, standard deviation, and floor and ceiling effects (percentage of patients with minimum and maximum theoretical scores, respectively). Small floor or ceiling effects (< 15%) are considered acceptable [21].

Two approximations were applied to estimate reliability: a) internal consistency assessed by Cronbach’s alpha coefficient [22]; and b) test-retest reproducibility evaluated by the intraclass correlation coefficient (ICC) [23]. Cronbach’s alpha coefficient ranges from 0 to 1 with values > 0.70 being considered acceptable [21]. An ICC of < 0.40 indicates poor to fair agreement, 0.41–0.60 moderate agreement, 0.61–0.80 good agreement and > 0.80 excellent agreement [24]. The PedsQL™ Oral Health Scale was re-administered between 2 and 4 weeks after the first administration in a sub-sample of 50% of the participants in each preschool, which were selected at random. Test-retest analysis was performed with this sub-sample, after excluding participants who reported change in oral health.

Confirmatory factorial analysis (CFA) was carried out to confirm the single dimension of the Chilean version proposed by developers of the PedsQL™ Oral Health Scale. For non-standardized solutions, the pattern of fixed and free factorial loads remained constant. Various fit indices were calculated to evaluate the model, including the Comparative Fit Index (CFI), the Tucker-Lewis Index (TLI) and the Root Mean Square Error Approach (RMSEA). CFI and TLI values greater than or equal to 0.95 indicate an excellent fit of the model, while values between 0.90 and 0.95 suggest only an acceptable fit of the model. RMSEA values below or equal to 0.06 indicate a good fit of the model, while values between 0.06 and 0.08 suggest only acceptable fit of the model [25].

Construct validity was assessed by following a known groups approach, comparing groups with presence or absence of oral conditions (caries, traumatic dental injuries and/or malocclusion) and groups defined by responses to the following questions: “In general, how would you rate the general health of your child?”, and “In general, how would you rate the dental health of your child?”. The possible answers to these two questions were: 1 = Excellent; 2 = Very good; 3 = Good; 4 = Fair; 5 = Poor. We hypothesized lower scores (poorer OHRQoL) in the PedsQL™ Oral Health Scale for the children whose parents reported regular or poor oral health; also, lower scores in the PedsQL™ Oral Health Scale among the groups of children with diagnosis of dental health problems in the oral examination. Comparisons between groups were performed with non-parametric Mann Whitney tests due to the score distributions of the PedsQL™ scales.

The convergent validity was assessed by examining the correlation between the PedsQL™ Oral Health Scale and ECOHIS, using Spearman’s correlation coefficients interpreted as follows: insignificant relation when r < 0.20; weak between 0.20 and 0.40; moderate between 0.40 and 0.60; moderate-to-strong between 0.60 and 0.80; and strong relation when r > 0.80 [26]. The convergent validity implies showing that different instruments measuring a similar concept present at least moderate correlations. We hypothesized that the correlation coefficients between ECOHIS and PedsQL™ Oral Health would be moderate-to-strong, since both were designed to measure OHRQoL. The data were analyzed using Stata 15 (Stata Corp, College Station, TX, USA).

## Results

### Transcultural adaptation

The translation of the 5 items was considered of low level difficulty (< 2.5). In the back-translation, the mean difficulty was 6.5 for item 3, and less than or equal to 3.5 for the rest. When the conceptual equivalence was compared between the back-translation and the original questionnaire, they were considered conceptually and linguistically equivalent in four of the five items. Only for item 3, “Having teeth that are dark in color”, the conceptual equivalence was rated B (conceptually equivalent, with grammatical differences): thus, after discussion by the group of researchers and translators, the expression in Spanish was modified to “Having some dark-colored teeth”. The cognitive interview showed that the instructions, items and response options were easily understood by the parents, and no problems were identified in distinguishing between the items or the different response options. All the parents agreed that the questions were intended to assess OHRQoL, and no modifications were needed as a result of the cognitive interviews.

### Study of metric properties

In total, 435 children aged between 2 and 5 years were contacted for the study, two of whom were excluded because they had special health needs; 12 parents declined to sign the informed consent form and 93 children did not attend the clinical examination. Of the 328 children examined, 26 parents did not reply to the questionnaire (response rate = 92.1%), and one questionnaire contained answers to fewer than 50% of the items and was therefore excluded from the analysis. Finally, a total of 301 families (children and parents) took part in the study. The descriptive information details are shown in Table 1. The mean age of the children included was 47.8 months (4 years) (SD = 13.3), 54% were boys (*n* = 162), and 75.7% presented low socio-economic status. Of the parents, 83.3% reported that their children had good or very good general health and 70.4% reported good or very good oral health. Seventeen percent declared that their children brushed their teeth once a day or less often, and 83.3% that the children brushed their teeth twice a day or more. The prevalence of caries experienced (dmft≥1) in the population examined was 53.8%, with a mean dmft of 2.52 (SD 3.71); the prevalence of malocclusions was 39.4%, and the traumatic dental injuries were 14.6%.

**Table 1:** Demographic and clinical characteristics of the children assessed in the study

Variables	*n* (%)
Child’s age in years (mean ± SD)	4.0 (1.1)
Child’s gender	
Male	162 (54.0)
Female	139 (46.0)
Socioeconomic status	
Low	228 (75.7)
Medium-high	73 (24.3)
Child’s general health, reported by parents	
Very good/Good	251 (83.3)
Regular/Poor	50 (16.6)
Child’s oral health, reported by parents	
Very good/ Good	212 (70.4)
Regular/ Poor	89 (29.5)
Tooth brushing	
Once a day or less	50 (16.6)
Twice or more	251 (83.3)
Simplified Oral Hygiene Index	
Good	161 (53.5)
Regular	125 (41.5)
Poor	15 (5.0)
Decayed, missing and filled teeth index (mean ± SD)	2,52 (SD 3.71)
Dental Caries	
Caries free (dmft = 0)	139 (46.1)
Caries presence (dmft > 1)	162 (53.8)
Malocclusion	
Absence	182 (60.6)
Presence	119 (39.4)
Traumatic Dental Injuries	
None	257 (85.4)
Infraction	27 (8.9)
Enamel fracture	4 (1.3)
Avulsion	2 (0.7)
Discoloration	11 (3.7)

All items of the PedsQL™ Oral Health Scale were completed by the participants, while 4.6% of them left some item without completion in the PedsQL™ 4.0 Generic Core Scale. The school functioning sub-scale reported the highest percentage of missing replies by the respondents (2.3%). The most reported problem was “having some dark-colored teeth” (32.6% almost never, sometimes, often or almost always) in the PedsQL™ Oral Health Scale; and for the PedsQL™ 4.0 Generic Core Scale, “[ …] has feeling angry been a problem for your child” (80.9% almost never, sometimes, often or almost always).

Table 2 shows distribution statistics and Cronbach’s alpha coefficient of the PedsQL™ scales. The mean scores in the PedsQL™ 4.0 Generic Core Scale and Oral Health Scale were 82.2 (SD = 12.2) and 89.1 (SD = 16.1) respectively. No floor effect was observed, but a substantial ceiling effect was observed in the PedsQL™ Oral Health Scale (46.5%). Cronbach’s alpha was 0.87 for the PedsQL™ 4.0 Generic Core Scale and 0.79 for the PedsQL™ Oral Health Scale. The ICC was 0.86 (95% CI = 0.78–0.91) for the PedsQL™ Oral Health Scale among the subsample of 84 parents who participated in the test-retest reliability study.

**Table 2:** Distribution and reliability of the PedsQL™ 4.0 Generic Core Scale and Oral Health Scale scores

Scale	Number of items	Observed range	Median (IQR)	Mean (SD)	Floor effect (%)	Ceiling effect (%)	Cronbach’s alpha
Total Generic Core Scale	21	42.9–100	84.5 (14.3)	82.2 (12.2)	0	2.66	0.87
Physical Functioning	8	18.8–100	90.6 (21.9)	85.27 (16.47)	0	26.6	0.83
Emotional Functioning	5	25–100	80 (25)	76.58 (16.27)	0	9.6	0.75
Social Functioning	5	20–100	90 (20)	85.99 (14.99)	0	32.9	0.74
School Functioning	3	16.7–100	77 (25)	76.95 (18.26)	0	21.3	0.56
Oral Health Scale	5	5–100	95 (15)	89.05 (16.08)	0	46.5	0.79

Floor effect: percentage of patients with minimum score; Ceiling effect: percentage of patients with maximum score

*IQR* Interquartile range; *SD* standard deviation

**Fig. 1:**
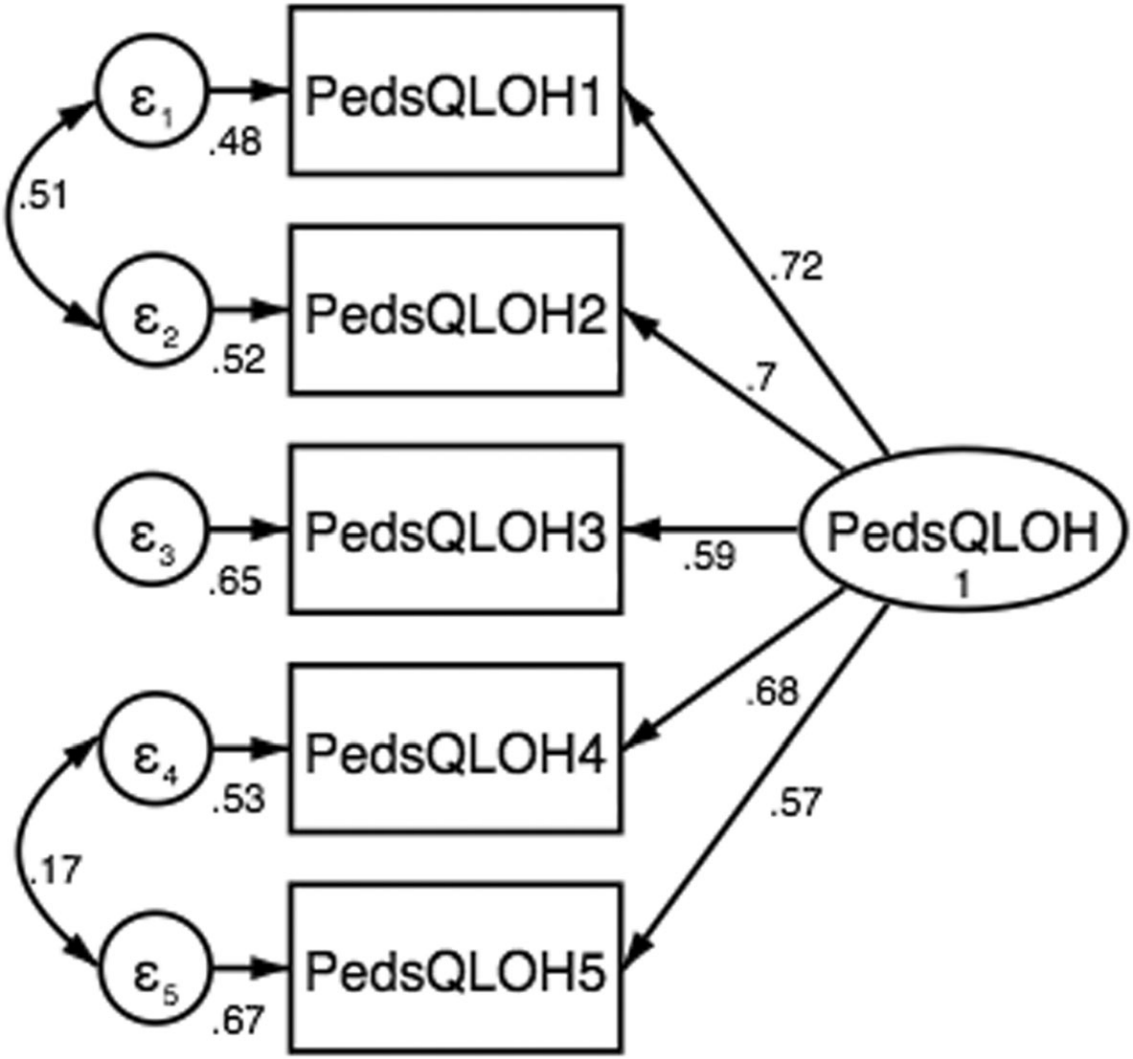
Confirmatory factor analysis on the 5-item PedsQL™ Oral Health Scale

Figure 1 shows results of the confirmatory factor analysis (CFA) in which the five items of the PedsQL™ Oral Health Scale loaded on a single latent variable. However, the CFA model did not fit the data adequately: CFI = 0.927, TLI = 0.855 and RMSEA = 0.160. To improve the overall model fit, a Lagrange multiplier test was applied and covariances were incorporated between: item 1 (Having tooth pain) and 2 (Having tooth pain when eating or drinking something hot, cold, or sweet); and item 4 (Having gum pain) and 5 (Having blood on his or her toothbrush after brushing). The new model showed a better goodness of fit indices: CFI = 0.996, TLI = 0.987 and RMSEA = 0.048.

Table 3 shows the results for the construct validity based on known groups. The children whose parents reported poorer general and oral health obtained significantly lower scores in the PedsQL™ Oral Health Scale, meaning a lower quality of life (*p* < 0.05). Finally, although the PedsQL™ Oral Health Scale score showed significant differences between children with and without dental caries (*p* < 0.0001), differences were no statistically significant between those with and without malocclusion (*p* < 0.142) or traumatic dental injuries (*p* < 0.126). An inverse moderate-to-strong (r = − 0.64) correlation between the PedsQL™ Oral Health Scale and ECOHIS was found, while the correlation with the generic core scale was moderate (r = 0.25).

**Table 3:** Construct validity of PedsQL™ Generic Core Scale and Oral Health Scale module based on known groups

Scale	Parents perception of child’s general health Median (IQR)		*p*	Parents perception of child’s oral health Median (IQR)		*p*	dmft Median (IQR)		*P*	Malocclusion Median (IQR)		*P*	TDI Median (IQR)		*p*
	Very good/Good	Regular/Poor		Very good/Good	Regular/Poor		0	> 1		Absence	Presence		Absence	Presence	
Physical Functioning	90.6 (21.9)	87.5 (31.3)	0.351	90.6 (18.8)	87.5 (28.1)	0.240	90.6 (21.9)	90.6 (25)	0.471	90.6 (21.9)	90.6 (25)	0.37	90.6 (21.9)	87.5 (18.8)	0.059
Emotional Functioning	80.0 (25)	75.0 (30)	0.032^a^	80.0 (20)	75 (25)	0.097	80.0 (20)	80.0 (25)	0.703	80.0 (25)	75 (25)	0.671	80 (25)	75 (27.5)	0.278
Social Functioning	90.0 (20)	90.0 (25)	0.555	90.0 (20)	90 (25)	0.607	90.0 (20)	90.0 (20)	0.605	90.0 (25)	90.0 (20)	0.54	90 (20)	85 (25)	0.437
School Functioning	83.3 (25)	75 (16.7)	0.063	83.3 (25)	75 (16.7)	0.206	77.0 (25)	77.0 (25)	0.489	83.3 (25)	75 (33.3)	0.167	77 (25)	75 (25)	0.278
Total Generic Core Scale	84.5 (15.4)	82.7 (21.4)	0.106	85.7 (15.5)	82.1 (20.2)	0.008^a^	83.3 (14.3)	85.4 (16.7)	0.454	85.4 (14.3)	84.5 (16.7)	0.334	85.7 (15.5)	81.5 (14.9)	0.227
Oral Health Scale	95.0 (15)	90.0 (25)	0.025^a^	100.0 (10)	85 (20)	< 0.001^a^	100.0 (5)	90.0 (25)	< 0.001^a^	97.5 (15)	95 (20)	0.142	95 (15)	90 (20)	0.126

^a^ Mann Whitney test *p* value < 0.05

*IQR* Interquartile range; *dmft* decayed, missing and filled teeth index; *TDI* Traumatic Dental Injuries

## Discussion

The Spanish version of the PedsQL™ Oral Health Scale toddler form for Chile presented high reliability and validity for measuring the parents’ perception of the quality of life related with their children’s oral health, when used together with the PedsQL™ 4.0 Generic Core Scale. The results are consistent with those obtained for the original PedsQL™ oral health module and suggest that the Chilean version is conceptually and metrically equivalent.

There was no data loss in the PedsQL™ Oral Health Scale, which was consistent with the findings of Bendo et al. [14]. These results suggest that the items making up the scale were well understood and acceptable for parents of pre-school children. In our study, none of the parents asked for help in filling out the questionnaires. The sub-scale which presented the highest non-response rate was school functioning. As the questionnaire was administered only to the parents, it is likely that they did not have enough information about their children’s development and behavior in preschool. Nevertheless, due to the cognitive immaturity and parental dependence of pre-school children, the parents are the best source for assessing their general and oral health [27].

The mean total scores of the PedsQL™ Oral Health Scale and the PedsQL™ 4.0 Generic Core Scale were quite high, indicating good perception by the parents of their children’s quality of life related with oral (mean 89 points) and general health (mean 82 points), similar to the study performed with the original scale (88 and 83 respectively). These good results are explained by the participants’ characteristics, since 54% of the children were free of oral conditions. Furthermore, systemic pathologies were considered exclusion criteria. We find these results surprising, as our sample was drawn from state schools attending low socio-economic population, and the study zone (District of Carahue) has a large rural component. However, various government programs have been implemented over the years to reduce the gaps existing in this population, so this is a promising result for further progress towards improving these strategies.

The high ceiling effect observed (46.5%) is similar to that reported by Bendo et al. [14] (37.5%), and consistent with the low number of oral problems experienced by our sample. Pakpour et al. [15] in contrast, detected a low ceiling effect (< 15%), which may be due to the fact that their study included older children (aged 8–18 years) with greater oral damage. For this reason, the Chilean version needs to be tested in populations with higher levels of oral problems to evaluate its metric properties among population with more severe oral problems.

The internal consistency of the PedsQL™ Oral Health Scale assessed by Cronbach’s alpha coefficient achieved the recommended standard of > 0.70. When the original version of this scale was evaluated in USA, it was 0.68. Our study presented slightly higher reliability values than the original study, which may be because we had a larger sample, allowing a more precise estimate. Pakpour et al. [15], in their adaptation and evaluation of the psychometric properties of these scales in an Iranian population, reported higher Cronbach’s alpha coefficient values than ours (0.89), with a considerably large sample (1053 children and 1026 parents) [15]. Bendo et al. [14], in a version adapted for Brazil, obtained Cronbach’s alpha coefficient values below the acceptable level (0.59). The test-retest reliability of the PedsQL™ Oral Health Scale in our study was high (0.86), similar to that reported in other validations (range 0.81–0.90) [15, 28, 29], indicating that the Chilean version presented good reproducibility when administered at two different times.

Our results of CFA supported that there was no discrepancy between observed values and the values expected under the theoretical model of the PedsQL™ Oral Health Scale. Similar results were previously reported after the incorporation of covariances in other studies carried out in schoolchildren from Brazil and Iran [14, 15]. No CFA has been reported by the original USA instrument.

The PedsQL™ Oral Health Scale was capable of distinguishing between groups defined by the dental state of the children, as reported by the parents. These findings were consistent with previous studies in which the parents who perceived that their children’s oral health was poor had significantly lower scores in the Oral Health Scale [14, 15]. Also, the score on the PedsQL™ Oral Health Scale was lower in children who presented dental problems than those suffering no disease (dmft = 0), which is consistent with studies carried out in Iran [15] and Brazil [14]. It is interesting to remark that those children whose parents reported worse oral health in their child presented a significantly worse emotional functioning, measured with the PedsQL™ 4.0 Generic Core subscale. The same was observed in the study of the Brazilian version [14] where PedsQL™ was administered to both parents and children.

The PedsQL™ Oral Health Scale was not able to measure the impact of malocclusion problems or traumatic dental injuries in our sample. A recent systematic review concluded that malocclusions in this age group are not perceived by the parents as a problem with impact on the child’s quality of life [1]. Furthermore, a meta-analysis showed that only very severe malocclusion could affect the overall OHRQoL [30]. The type of traumatic dental injuries detected most frequently in our study was enamel fracture, which does not have a significant impact on the quality of life of pre-school children [3]. It is important to consider that the prevalence of TDI was low (14.5%) in our sample and only 2 preschoolers suffered complicated TDI (avulsion). On the other hand, although differences per malocclusion or TDI were not statistically significant due to its small magnitude, in both cases the patients without these problems showed a better OHRQoL than those with them. Studies based in schools have the advantage of including children with a wide range of good and poor oral health, and the disadvantage of including less severe oral problems than in clinical settings.

As hypothesized a priori, the correlation between the PedsQL™ Oral Health Scale and ECOHIS was high, since their items are related principally with dental pain [31]. Both are specific scales designed to measure the same construct, OHRQoL. Although moderate, the correlation found with the PedsQL™ Generic Core Scale was lower than reported in other studies [14, 15]. Generic HRQoL instruments are unable to measure the impact of the small but important impairment produced for a particular pathology. Diseases may affect different functions and lead to different physical or emotional problems, or affect other aspects of quality of life [32]. Our results are in line with other studies which show that OHRQoL scales are more sensitive than the PedsQL™ Generic Core Scale in measuring the impact of oral problems in pre-school children [31].

One of the main limitations of the study was that the sample was drawn from a location in southern Chile with high indices of low income and rural residence. It was therefore quite homogeneous in socio-economic terms. In addition, being a community-based sample, not all conditions were well represented. Therefore, we cannot recommend yet the use of the Chilean version of the PedsQL™ Oral Health Scale to measure the impact of TDI or malocclusions on OHRQoL. Further research is needed in other settings to explore the ability of the instrument to discriminate between certain degrees of these conditions. However, this is a priority population for the implementation of public policies, and evaluation of their OHRQoL can provide information to support decision-making. Although the PedsQL form for toddlers was developed for the age of 2–4 years, we decided to include children up to and including five years old because we needed a tool that can be used in preschoolers (2–5 years old). On the other hand, it is relevant to highlight that the parent-reported forms are the same for each specific age group. Furthermore, the sensitivity to change was not assessed, so further studies are needed to assess the ability of the Chilean version of the PedsQL™ Oral Health Scale to detect changes over time.

## Conclusions

The results of the present study support the applicability, reliability and validity of the Spanish language version of the PedsQL™ Oral Health Scale for Chilean population, reported by the parents or guardians of children aged 2 to 5 years. Comparison with the metric properties study of the original instrument showed similar results for validity and reliability, supporting equivalence between this cross-cultural adaptation and the original PedsQL™ Oral Health Scale for toddlers.

## Data Availability

The datasets used and/or analyzed during the current study are available from the corresponding author upon reasonable request.

## Abbreviations

dmft: Decayed, missing and filled teeth index
ECOHIS: AEarly Childhood Oral Health Impact Scale
ICC: Intraclass correlation coefficient
OHRQoL: Oral Health-Related Quality of Life
PedsQL™4.0: Pediatric Quality of life Generic Core scale

## References

[1] Kragt L, Dhamo B, Wolvius EB, Ongkosuwito EM (2016). The impact of malocclusions on oral health-related quality of life in children-a systematic review and meta-analysis. Clin Oral Invest.

[2] Scarpelli AC, Paiva SM, Viegas CM, Carvalho AC, Ferreira FM, Pordeus IA (2013). Oral health-related quality of life among Brazilian preschool children. Community Dent Oral Epidemiol.

[3] Zaror C, Martinez-Zapata MJ, Abarca J, Diaz J, Pardo Y, Pont A, Ferrer M (2018). Impact of traumatic dental injuries on quality of life in preschoolers and schoolchildren: a systematic review and meta-analysis. Community Dent Oral Epidemiol.

[4] Sheiham A (2006). Dental caries affects body weight, growth and quality of life in pre-school children. Br Dent J.

[5] Gift HC, Reisine ST, Larach DC (1992). The social impact of dental problems and visits. Am J Public Health.

[6] Ladrillo TE, Hobdell MH, Caviness AC (2006). Increasing prevalence of emergency department visits for pediatric dental care, 1997-2001. J Am Dent Assoc.

[7] Sischo L, Broder HL (2011). Oral health-related quality of life: what, why, how, and future implications. J Dent Res.

[8] Allen PF (2003). Assessment of oral health related quality of life. Health Qual Life Outcomes.

[9] McGrath C, Broder H, Wilson-Genderson M (2004). Assessing the impact of oral health on the life quality of children: implications for research and practice. Community Dent Oral Epidemiol.

[10] Zaror Carlos, Pardo Yolanda, Espinoza-Espinoza Gerardo, Pont Àngels, Muñoz-Millán Patricia, Martínez-Zapata María José, Vilagut Gemma, Forero Carlos G., Garin Olatz, Alonso Jordi, Ferrer Montse (2018). Assessing oral health-related quality of life in children and adolescents: a systematic review and standardized comparison of available instruments. Clinical Oral Investigations.

[11] Steele MM, Steele RG, Varni JW (2009). Reliability and validity of the PedsQL™ Oral health scale: measuring the relationship between child oral health and health-related quality of life. Child Health Care.

[12] Varni James W., Seid Michael, Rode Cheryl A. (1999). The PedsQL™: Measurement Model for the Pediatric Quality of Life Inventory. Medical Care.

[13] Plaza M (2005). Calidad de vida de los niños hemofilicos de 8 a 12 años de la región Metropolitana de Santiago de Chile.

[14] Bendo CB, Paiva SM, Viegas CM, Vale MP, Varni JW (2012). The PedsQL Oral health scale: feasibility, reliability and validity of the Brazilian Portuguese version. Health Qual Life Outcomes.

[15] Pakpour AH, Yekaninejad MS, Zarei F, Hashemi F, Steele MM, Varni JW (2011). The PedsQL Oral health scale in Iranian children: reliability and validity. Int J Paediatr Dent.

[16] Wild D, Grove A, Martin M, Eremenco S, McElroy S, Verjee-Lorenz A (2005). Principles of good practice for the translation and cultural adaptation process for patient-reported outcomes (PRO) measures: report of the ISPOR task force for translation and cultural adaptation. Value Health.

[17] Petersen PE, Baez RJ, World Health Organization. Oral health surveys: basic methods. 5th ed. Geneve: World Health Organization; 2013. https://apps.who.int/iris/handle/10665/97035.

[18] Andreasen J, Andreasen F (1994). Classification, etiology adn epidemiology. Texbook and color atlas of traumatic injuries to the teeht.

[19] Abanto J, Carvalho TS, Mendes FM, Wanderley MT, Bonecker M, Raggio DP (2011). Impact of oral diseases and disorders on oral health-related quality of life of preschool children. Community Dent Oral Epidemiol.

[20] Streiner DL, Kottner J (2014). Recommendations for reporting the results of studies of instrument and scale development and testing. J Adv Nurs.

[21] Terwee CB, Bot SD, de Boer MR, van der Windt DA, Knol DL, Dekker J (2007). Quality criteria were proposed for measurement properties of health status questionnaires. J Clin Epidemiol.

[22] Streiner DL (2003). Starting at the beginning: an introduction to coefficient alpha and internal consistency. J Pers Assess.

[23] Prieto L, Lamarca R, Casado A (1998). Assessment of the reliability of clinical findings: the intraclass correlation coefficient. Med Clin (Barc).

[24] Bartko JJ (1996). Intraclass correlation coefficient as a measure of reliability. Psychol Rep.

[25] Hu LT, Bentler PM (1999). Cutoff criteria for fit indices in covariance structure analysis: conventional criteria versus new alternatives. Struct Equ Model.

[26] Franzblau A (1958). Correlation coefficients. A primer of statistics for non-statisticians.

[27] Eiser C, Mohay H, Morse R (2000). The measurement of quality of life in young children. Child Care Health Dev.

[28] Bendo CB, Paiva SM, Varni JW, Vale MP (2014). Oral health-related quality of life and traumatic dental injuries in Brazilian adolescents. Community Dent Oral Epidemiol.

[29] Lin CY, Kumar S, Pakpour AH (2016). Rasch analysis of the Persian version of PedsQL (TM) Oral health scale: further psychometric evaluation on item validity including differential item functioning. Health Promot Perspect.

[30] Sun L, Wong HM, McGrath CPJ (2018). Association between the severity of malocclusion, assessed by occlusal indices, and oral health related quality of life: a systematic review and meta-analysis. Oral Health Prev Dent.

[31] Lee GH, McGrath C, Yiu CK, King NM (2010). A comparison of a generic and oral health-specific measure in assessing the impact of early childhood caries on quality of life. Community Dent Oral Epidemiol.

[32] Guyatt GH, Bombardier C, Tugwell PX (1986). Measuring disease-specific quality of life in clinical trials. CMAJ..

